# The Impact of Cancer Coalitions on the Dissemination of Colorectal Cancer Materials to Community Organizations in Rural Appalachia

**Published:** 2006-03-15

**Authors:** Ann J Ward, Brenda Coffey Kluhsman, Eugene J Lengerich, Andrea M Piccinin

**Affiliations:** Community Cancer Control, Research, and Education; Ms. Ward is also affiliated with the Department of Food Science, Pennsylvania State University College of Agricultural Sciences, University Park, Pa; Department of Health Evaluation Sciences, Pennsylvania State University College of Medicine, Hershey, Pa; Department of Health Evaluation Sciences, Pennsylvania State University College of Medicine, Hershey, Pa; Department of Statistics, Pennsylvania State University Eberly College of Science, University Park, Pa

## Abstract

**Introduction:**

The incidence of colorectal cancer in portions of rural Appalachia is higher than in much of the United States. To reduce this disparity, cancer-control strategies could be adapted to and implemented in rural Appalachian communities. The objectives of this pilot study were to develop and test community-based participatory research methods to examine whether cancer coalitions in Appalachia could effectively disseminate print materials from a national media campaign intended to promote colorectal cancer awareness to their rural communities.

**Methods:**

This pilot study used a two-arm intervention design with random selection of 450 community organizations from nine counties with cancer coalitions (the coalition arm) and 450 organizations from nine matched counties without a cancer coalition (the noncoalition arm) in northern Appalachia. The primary outcome measures were participation by and interest of community organizations in dissemination of materials from *Screen for Life:* *National Colorectal Cancer Action Campaign*, a national campaign to promote colorectal cancer education and screening. The data were collected with prestudy and poststudy surveys.

**Results:**

One-hundred thirty (29%) organizations participated in the coalition arm, and 38 (8%) participated in the noncoalition arm (*P* < .001). Within the coalition arm, 86 of the 119 (66%) organizations that responded to the question about influence reported being influenced to participate by the local coalition. Initial interest in dissemination was high in each of the study arms but remained higher throughout the study in the coalition arm than the noncoalition arm.

**Conclusion:**

Community cancer coalitions can increase the local dissemination of material from a national media campaign in rural Appalachia. Continued development and study of methods for coalitions to translate and implement cancer-control strategies at a local level in Appalachia is warranted.

## Introduction

The incidence of colorectal cancer in portions of rural Appalachia has been found to be higher than in much of the United States (1). To reduce this disparity, rural Appalachian communities will need to adapt evidence-based cancer-control strategies for their unique environments, in which barriers include limited access to health care services, low incomes, low literacy levels, lack of health insurance, high unemployment, and other social and economic factors that impede communication (2-4).

The systematic process by which new information or strategies are adopted by members of a social system is referred to as *diffusion of innovation* (5-6). Multiple channels, including mass media and interpersonal communication, can facilitate the process; however, communicating new cancer-control strategies, including diffusing information, is difficult in unique geographic regions such as rural Appalachia.

In the previous decade, policymakers, program planners, and funding agencies relied more on community coalitions to communicate cancer-control strategies (7-9). For example, coalitions have encouraged cancer screenings and health promotion campaigns and have distributed educational materials to schools, libraries, and other community settings (4-5). In addition to the actions of the entire coalition, individuals within a coalition may encourage communication by being early adopters, advocates of innovations, or *linking agents* of new cancer-control strategies (10-13). *Linking agent* refers to the connection between the source of an innovation and the ultimate adopter: "The linking agent may be a public or private entity but has a primary role in making personal contacts, transmitting information, and actively advocating target innovations to service delivery agencies" (13). If trusted by the community, the actions of such opinion leaders may encourage other individuals in the community to adopt the cancer-control strategy or information.

Thus, a community cancer coalition may be a linking agent in the dissemination of new strategies or information from national and state public health partners to the local community. The degree to which coalitions may be linking agents for dissemination in rural Appalachia has not been fully investigated (12). If found to be effective in diffusion of cancer-control strategies or information, community cancer coalitions may be an important component of national efforts to reduce the incidence of cancer, particularly in geographic areas that lack many resources for cancer control.

Community-based participatory research (CBPR) conducted by coalitions in partnership with academic and clinical investigators is a viable approach for examining the role of community coalitions in the diffusion of cancer-control strategies. The premise of CBPR is that by sharing unique strengths, knowledge, resources, decision making, trust, power, responsibilities, and ownership in a project, community stakeholders and research partners will develop a better understanding of a public health issue and the communities' needs and will more likely notice improved health outcomes through the resulting research (8,14-19). In addition, CBPR efforts tend to yield a deeper understanding of communities' resources, culture, and disparities, allowing research partners, such as cancer coalitions, to more effectively adapt best practices to their communities' needs (19). CBPR has been used previously for cancer-control research (5,20-30).

The objective of our pilot CBPR study, which was implemented through a collaboration between university investigators and community members, was to examine whether cancer coalitions in rural Appalachia could be linking agents to the community in the dissemination of print materials from a national media campaign intended to promote colorectal cancer education and screening awareness. We hypothesized that organizations that received campaign materials directly from a coalition would have greater participation and interest in disseminating the materials than community organizations that received the materials from a university. In addition, the study sought to examine the impact of an organization's type, size, and previous cancer-control experience on participation levels in dissemination.

### Screen for Life: National Colorectal Cancer Action Campaign

*Screen for Life: National Colorectal Cancer Action Campaign* is a national, multiyear, multimedia campaign to inform people aged 50 years and older about the importance of having regular colorectal cancer screening tests (31). Launched in 1999, *Screen for Life* was designed, developed, and implemented by the Centers for Disease Control and Prevention (CDC) and the Centers for Medicare and Medicaid Services (formerly the Health Care Financing Administration), with technical support from the National Cancer Institute (NCI). *Screen for Life* campaign messages and materials, including brochures, posters, and public service announcements for radio and television, were developed after formative research, including an extensive review of published communication and behavioral science literature and more than 100 focus groups of men and women aged 50 years and older conducted in more than 40 U.S. cities.

### The Appalachia Cancer Network

The Appalachia Cancer Network (ACN), one of 18 projects in the NCI-funded Special Populations Network for Cancer Awareness, Research, and Training, is a consortium of academic institutions, cancer centers, departments of health, cancer advocacy organizations, community groups, and volunteers addressing cancer-control issues through education and research in eight Appalachian states. The Northern Appalachia Cancer Network (NACN), which is based at the Pennsylvania State University (Penn State), works with 11 community cancer coalitions composed of 187 active individuals, of whom 102 belong to state and local community organizations in rural Pennsylvania and New York. Most of the coalitions have been active in cancer-control programs and have been working with the study investigators since 1993. Coalition members include unaffiliated volunteers (45%) and members of cancer organizations and support groups (11%), health care organizations (19%), community human service organizations (11%), county and district health departments (7%), and educators from Penn State's and Cornell University's cooperative extensions (6%).

In 2000, Pennsylvania ranked sixth and New York ranked eleventh highest nationally for colorectal cancer incidence (Pennsylvania, 60.4 per 100,000; New York, 58.7 per 100,000), exceeding the U.S. rate of 54.6 per 100,000 (32). In addition, in 2002, approximately half of Pennsylvanians (52%) and New Yorkers (49%) aged 50 years and older had never received a sigmoidoscopy or colonoscopy (33).

## Methods

### Study design

To test the study hypothesis, we implemented a two-group pilot intervention study in March 2003 using a prestudy–poststudy design with random selection of community organizations (Figure). We also included a follow-up survey of organizations that chose not to join the study. The primary outcome measures were community organizations' participation and interest levels in disseminating the materials to their clients and employees. Using a CBPR approach, university investigators and community cancer coalition members collaborated to conduct the research. In addition, the protocol was designed to standardize data collection procedures yet permit flexibility and creativity so that the coalitions could tailor their mode of interactions with community organizations to the unique sociocultural environment of their counties. Bimonthly phone calls with university investigators and weekly calls and monthly meetings with field staff members were used to communicate with coalitions and project leaders. During these meetings, the protocol was discussed and adaptations to the intervention were implemented. The study was approved by the institutional review boards of Penn State University and Penn State–Milton S. Hershey Medical Center.

**Figure:**
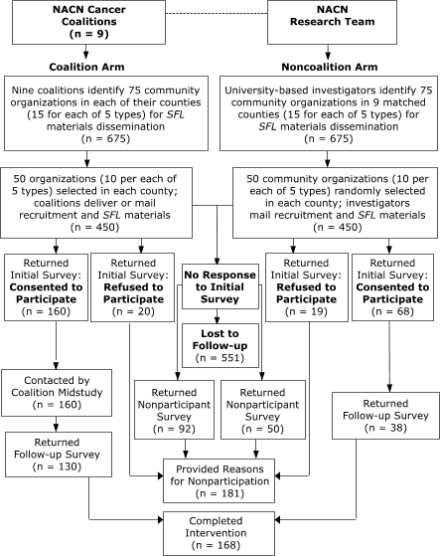
Dissemination of materials by community cancer coalitions to community organizations, Northern Appalachia Cancer Network (NACN) *Screen for Life (SFL) *Pilot Study, 2003.

**Table:** 

The figure is a flow chart explaining the participation and nonparticipation of organizations in the study, by cancer coalition arm or by noncoalition arm. Of the NACN cancer coalitions (on the left side of the flow chart), nine coalitions identified 75 community organizations in each of their counties (15 for each of 5 types) for SFL materials dissemination (n = 675). Fifty organizations (10 per each of 5 types) were selected in each county; coalitions delivered or mailed recruitment and SFL materials (n = 450). Of the 450, 160 returned the initial survey and consented to participate; 20 returned the initial survey and refused to participate but provided reasons for nonparticipation. Of the 160 that consented, all were contacted by the coalition midway through the study, and 130 of them completed the follow-up survey.
On the right side of the flow chart, of the NACN research team, noncoalition arm, the university-based investigators identified 75 community organizations in 9 matched counties (15 for each of 5 types) for SFL materials dissemination (n = 675). Fifty community organizations (10 per each of 5 types) were randomly selected in each county; investigators mailed recruitment and SFL materials (n = 450). Of the 450, 19 returned the initial survey and refused to participate but provided reasons for nonparticipation. Of the 450, 68 returned the initial survey and consented to participate (n = 68). Of the 68 that consented, 38 returned the follow-up survey.
Of the original 900 (450 in the coalition arm and 450 in the noncoalition arm), 551 were lost to follow up. Of the 900, 181 did not participate but provided reasons for nonparticipation, and 168 completed the intervention.

### Coalition arm: NACN coalitions as research partners

By 2002, the 11 NACN coalitions had begun to address the burden of colorectal cancer in the region through implementation of 143 colorectal cancer interventions (NACN Coalition Database, unpublished data). In 2003, university researchers invited the NACN coalitions to participate in the study as research partners. Personal invitations to work on the study were described in a project fact sheet and timeline and provided by NACN field staff at monthly coalition meetings.

NACN coalitions are unique entities that vary in size and composition but share the common goal of reducing the cancer incidence in their rural communities. At the time of this study, the median coalition size was 12 members (with a range of 9 to 24), and all but one coalition had a mixture of volunteer and agency members (median, 20% volunteers; range, 0% to 55%). The coalitions also varied by primary cancer focus, with most addressing breast and cervical cancer screening but many also addressing colorectal, prostate, and skin cancer prevention and tobacco-control issues.

Investigators requested an 8-month commitment, of which 65 hours per coalition were anticipated for the project. Nine of the 11 NACN coalitions volunteered to participate in the study. Each coalition recruited a *Screen for Life* project subcommittee and chairperson. The chairperson was trained and approved for human subjects research through the institutional review board of the university. Members from each subcommittee worked with university investigators to refine the research protocol time frame and create a partnership agreement, which all parties signed. Key points in the agreement included the specific roles and responsibilities of the coalitions and university researchers, communication expectations, time commitments and resources to be provided by both parties, joint participation in project evaluation, plans for dissemination of results, and the flexibility of coalitions to tailor their approaches to the organizations in the study.

### Noncoalition arm: matched counties

For the noncoalition arm, each county without a cancer coalition was matched to one of the home counties of the nine participating coalitions (Table 1) (34-37). The matching counties were selected based on state (Pennsylvania or New York), Appalachia county designation, and the unweighted average of the closeness ranks calculated from seven demographic criteria: population size, population density, poverty rate, population per primary care provider, age, education, and rurality.

### Eligibility, identification, and selection of community organizations

Community organizations were considered eligible if they were able to provide *Screen for Life* materials to the adult public (i.e., employees or clients) and had a physical facility in the county from which the materials could be distributed. To understand reasons for participation and how perceived benefits may have differed by type of organization, selection of the organizations was stratified into five groups:

1. Civil, fraternal, and service (e.g., human service agencies, service clubs, libraries, police departments, government agencies)
2. Health care (e.g., pharmacies, physicians' offices, health clinics, hospitals, family planning clinics)
3. Aging (e.g., senior centers, assisted living facilities, senior apartments)
4. Business (e.g., retail stores, restaurants, factories, schools and universities, banks, post offices)
5. Religious (e.g., churches, temples, mosques)

To identify potential community organizations as study participants, NACN investigators and coalitions created a list of 75 eligible organizations (15 in each of the five organization groups) in 18 counties for a total of 1350 organizations. For the coalition arm, each coalition identified the potential organizations by reviewing lists from local human service agencies, departments of health, hospital associations, and other community sources. Seven coalitions also used the consumer health profiles from the NCI's Cancer Information Service (38) to identify geographic areas in their home counties that were medically underserved. For the noncoalition arm, NACN investigators published lists similar to those in the coalition counties; because they lacked local coalition contacts, they also used an online information service (39) to identify potential community organization study participants.

Investigators randomly selected organizations by first alphabetizing the organization lists and then choosing every seventh organization without replacement until 10 organizations for each of the five types of organizations were selected for each county. The final sample size was 900 (450 organizations per arm).

### Data collection

Organizations in the final sample received a recruitment package with an invitation letter, human subjects information, the initial survey, a sample *Screen for Life* brochure (*Let's Break the Silence,* available from www.cdc.gov/cancer/screenforlife/fb_silence.htm) and poster (*True or False,* available from www.cdc.gov/cancer/screenforlife/trueorfalse.htm), a county fact sheet on colorectal cancer and screening guidelines, and a stamped, preaddressed return envelope.

Invitation methods and materials for each arm of the study were identical except that in the coalition arm, invitation materials were printed on coalition letterhead, included personal contact information for the coalition, and were mailed or hand delivered by coalition members to identified community organizations. In the noncoalition arm, invitation materials were printed on NACN letterhead, included investigator contact information, and were mailed by NACN investigators to identified organizations. Invitation materials in each arm were customized with county-specific colorectal cancer data.

The initial survey requested information about the organization, its previous experience with distributing colorectal and other cancer information, and previous collaboration with cancer-control organizations. The survey also requested information on the number of employees and weekly visitors to or customers of the organization to determine whether organization size might be related to participation. A 4-point Likert scale (not at all, not very, somewhat, very) was used to rate organizations' interest in and importance placed on 1) dissemination of *Screen for Life* materials, 2) promotion of colorectal cancer screening awareness, 3) health promotion related to other cancers, and 4) general health promotion. In addition, reasons for participation in the study (or for declining participation) were elicited.

Organizations that returned the initial survey and reported that they were willing to distribute the *Screen for Life* materials (referred to as *participating organizations*) subsequently received 35 *Screen for Life* brochures and three posters for distribution to their employees and clients. In the coalition arm, coalition members mailed or hand delivered the materials and recontacted the participating organizations 2 weeks later (midstudy) to determine whether and how the materials were used. If requested by the organization, coalition members provided additional *Screen for Life* materials to the organization for distribution. In the noncoalition arm, NACN investigators mailed the *Screen for Life* materials to the identified organizations, with no midstudy follow-up contact from NACN investigators.

Four weeks after the initial mailing, participating organizations in both arms were mailed the final survey. As mentioned, the survey collected information on the perceived importance of distribution of colorectal cancer awareness information, benefits of participation, and interest in dissemination of cancer information in the future. In addition, organizations in the coalition arm were asked to what extent a call or visit from a coalition member influenced their participation in the study and how interested the organization was in working with the coalition in the future. To increase the response rate in the coalition counties, coalition members made follow-up telephone calls to participating organizations that had not returned the final survey. In the noncoalition arm, university investigators mailed a reminder post card. In addition, organizations that declined to participate at any stage of the study were mailed a brief survey asking why they had decided not to participate.

### Data analysis

Descriptive statistics were used to provide frequencies and ranges of values for the initial and follow-up surveys. Chi-square analysis of contingency tables was conducted with follow-up comparisons of significant tables larger than 2 × 2. An α level of .05 was used for all statistical tests except for the follow-up tests, when α was adjusted according to Bonferroni's correction.

## Results

Of the 900 organizations contacted, 228 (25%) completed and returned the initial survey, 160 (36%) in the coalition arm, and 68 (15%) in the noncoalition arm (Table 2). Of the 228 that completed the initial survey, 168 (74%) subsequently returned the follow-up survey, for an overall participation rate of 19%. A significantly larger number of organizations that participated in the study were from the coalition arm rather than the noncoalition arm (*P* < .001). With the exception of analysis of reasons for nonparticipation, the following results are for the organizations that completed both the initial and follow-up surveys.

For the two study arms combined, participation was significantly associated with type of organization (*P* < .001) (Table 3). Civic, service, and fraternal organizations (29%) were more likely to participate than were business (16%) or religious organizations (8%) (*P* < .001 for each comparison). Participating organizations ranged in size (total number of employees and estimated number of weekly visitors) from 2 to 15,063. After removing from analysis the two largest organizations, no association between organization size and participation level was found (*P = .*78).

In the coalition arm, a phone call or visit from a coalition member was reported to have strongly or somewhat influenced the decision to participate for 86 organizations (Table 4). However, the effect of coalition contact on participation did not differ significantly by organization type (*P* = .055).

### Organizations' experiences and reasons for participation

Of the 168 organizations in both study arms, 73 (44%) reported no previous experience in cancer control (data not shown). Among organizations with such experience, 55 (33%) reported experience with other cancer-control activities but not with colorectal cancer materials, and an additional 28 (17%) had previous experience with colorectal cancer but not *Screen for Life* materials. Only 12 (7%) had prior experience with *Screen for Life*. Organizations in the coalition study arm had significantly greater previous cancer experience than those in the noncoalition arm (*P = .*009). Health care organizations had significantly greater previous cancer experience than did other organization types (*P* < .001).

The overall distribution of reasons for dissemination was similar for the two study arms. The most commonly reported reasons for dissemination (with multiple reasons available for selection) were to promote the health of the community (71%), promote the health of the organization's clientele (64%), and promote the health of the organization's members (59%) (Table 5). Compared with other organizations (except for aging organizations), health care organizations were more likely to participate to promote the health of their clientele (*P* < .001) because it was the mission of the organization (*P* < .001) and because of their organization members' experience with cancer (*P =* .01). The frequency and distribution of reported benefits of dissemination of *Screen for Life* materials were similar to the frequency and distribution of reasons for participation (data not shown).

### Interest in and importance of dissemination

Overall, participants ranked initial interest in and importance of dissemination high (Table 6). Initial interest did not differ between study arms (*P = .*30), whereas initial level of importance was greater among organizations in the coalition arm than in the noncoalition arm (*P = .*03). Initial interest levels (*P = .*02) and initial importance levels differed significantly (*P = .*002) between organization types in the coalition arm. Business organizations reported the lowest level of initial interest and importance but also the greatest increase in both during the study.

Of the organizations that reported data for both surveys, 91% increased or maintained initial levels of interest, whereas 92% increased or maintained initial levels of importance. Change in interest level was greater among organizations in the coalition arm than in the noncoalition arm (*P* < .001) and differed significantly between organization types in the coalition arm (*P = .*01). Change in importance did not differ significantly between study arms (*P = .*95) but did differ significantly between organization types in the coalition arm (*P = .*006).

A significantly greater proportion of organizations that had previous experience with cancer control maintained or increased their interest in dissemination of *Screen for Life* materials than did organizations without previous experience (*P = .*05) (data not shown). During the course of the study, religious organizations decreased their level of interest and importance in the coalition arm.

### Nonparticipating organizations

Reasons for nonparticipation in the study were requested in the initial survey and in a short follow-up survey mailed to nonresponders. In total, 181 nonparticipating organizations returned the initial surveys (39 organizations) and follow-up surveys (142 organizations). Of these, 134 (74%) organizations reported 218 reasons for not participating. The most frequently reported reasons for nonparticipation included lost or misplaced study materials (17%), inadequate resources to disseminate the materials (16%), and misunderstanding of the project's expectations (13%). However, 24 (13%) of the nonparticipating organizations reported that they would like to participate in a similar study in the future. In addition, 11 (6%) nonparticipating organizations used the sample *Screen for Life* materials, mailed with the recruitment survey, despite having declined to formally participate in the study.

## Discussion

Dissemination of *Screen for Life* materials by community organizations in rural Appalachian counties of Pennsylvania and New York was significantly greater when a community cancer coalition delivered the intervention than when university investigators delivered the intervention. Approximately 75% of 130 organizations in the coalition arm of the study reported being positively influenced to participate by the presence of the coalition. The findings from this study support the hypothesis that community cancer coalitions are effective linking agents that can increase the dissemination of colorectal cancer-control strategies to community organizations (10-13) in rural Appalachia that might not have any other way to access the strategies.

We also found differences among types of participating organizations. Civic, service, and fraternal organizations were more likely to disseminate the materials, and religious organizations were the least likely to disseminate them. Whereas few community organizations had previous experience with *Screen for Life,* health care organizations were more likely to report previous experience in cancer control, and business organizations were least likely to report such experience. Health care organizations had slightly different reasons for disseminating the *Screen for Life* materials. Business organizations had the lowest initial level of interest and importance, whereas the level of interest and importance from religious organizations was more likely to decrease during the study. These findings suggest that efforts and messages to promote dissemination of cancer-control innovations might be tailored to the specific type of organization.

As part of our community-based participatory approach, the coalitions contributed substantially to the design, implementation, and strengths of this study. Coalition members identified communication channels and strategies to effectively reach local organizations. They made phone calls, visited organizations, resent surveys, and motivated organizations to disseminate materials and return surveys. The volunteer research partners also showed an exemplary commitment to the research protocol, with attention to quality and detail at a level expected of professional researchers. In the final survey, 100 (60%) of the participant organizations in the coalition arm stated that they would like to work with the coalitions in the future or receive additional cancer information resources from them. These findings suggest that CDC can extend the distribution of *Screen for Life* materials into rural organizations and communities through cancer coalitions.

Our study was limited by numerous factors. First, the overall participation rate was 19%, with 29% participation in the coalition arm and 8% in the noncoalition arm. Little evidence exists to explain the low overall participation rate. A review of the scientific CBPR literature, which was commissioned in 2001 by the Agency for Healthcare Research and Quality, revealed that contact with community members generally raised participation rates in 8 of 12 completed intervention studies (19). Reasons stated by respondents for not participating suggest that some members of community organizations may not have perceived a need for colorectal cancer control, did not remember receiving the study materials, or lacked the resources needed to participate. In addition, in counties where coalitions helped to identify organizations, participation may have been biased toward coalition-friendly organizations; thus, it is possible that the positive coalition effect was overestimated. More research is needed to determine factors associated with participation rates in community-based participatory studies.

A second possible source of bias may have been in the study design, specifically the midstudy contact. In national media campaigns, the distribution of materials tends to be impersonal, as it was in the noncoalition arm; the organizations received mailed materials from a central source for community dissemination and had no personal contact. In the coalition arm, dissemination was more personalized, and coalition members chose to follow up with organizations by telephone or in person for the midstudy contact. Third, the duration of the study, which was chosen to coincide with National Colorectal Cancer Awareness Month, was short and limited the time we had to work closely with the coalitions. A longer time frame would have allowed earlier coalition involvement in the intervention planning and may have resulted in greater dissemination of *Screen for Life* materials. Fourth, different individuals in the organization may have responded to the initial and follow-up surveys because our protocol did not require the same person to respond to each survey. Finally, the ability to generalize these findings may be limited because the study was conducted in rural Appalachian counties in Pennsylvania and New York only.

Despite these limitations, we found evidence that personal contact, whether by telephone or in person, from members of community-based cancer coalitions can increase dissemination of materials from the *Screen for Life* campaign to diverse community organizations. Strategies to garner the participation of community organizations may need to be tailored to the type of organization involved. In addition, community cancer coalitions can significantly contribute to the process of cancer-control research in rural Appalachia. This study demonstrates that community cancer coalitions can contribute to the adoption of evidence-based strategies for colorectal cancer control, which ultimately may reduce the high colorectal cancer incidence in rural Appalachian communities.

## Figures and Tables

**Table 1:** Characteristics of Study Counties by Study Arm, Northern Appalachia Cancer Network *Screen for Life* Pilot Study, 2003

**Characteristic**	**Coalition Arm**		**Noncoalition Arm**	

	**Median**	**Range**	**Median**	**Range**
Population size^a^	83,382	37,546-139,750	51,401	41,765-152,598
Population density (no. people/sq mile)^a^	108	33-263	71.1	49-222
Poverty rate (% below poverty level)^a^	12.5	9.9-17.3	11.8	7.7-15.40
Population per primary care provider^b^	2240	1651-3120	2223	1017-2907
Age (% older than 65 y)^a^	16.0	13.3-19.3	16.5	12.3-19.7
Education (% older than 25 y with high school diploma)^a^	47.6	45.3-81.2	50.7	47.4-82.8
Rurality (Beale codes)^c^	4	2-7	4	1-7

a Source: data from United States Census of Population and Housing 2000 (35)

b Source: data from Find a Health Professional Shortage Area (36)

c Source: data from Rural-Urban Continuum Codes (37)

**Table 2:** Participation in Dissemination of Materials, by Study Arm, Northern Appalachia Cancer Network *Screen for Life* Pilot Study, 2003

**Survey**	**Total (Both Arms)**			**Coalition Arm**			**Noncoalition Arm**			***P Value***

	**Contacted, No.**	**Respondents, No.**	**%**	**Contacted, No.**	**Respondents, No.**	**%**	**Contacted, No.**	**Respondents, No.**	**%**
Initial survey	900	228	25.3	450	160	35.6	450	68	15.1	<.001^b^
Follow-up survey	228	168	73.7	160	130	81.3	68	38	55.9	<.001^c^
Total^a^	—	—	18.7	—	—	28.9	—	—	8.4	<.001^d^

a Resulting participation percentages include organizations that completed both initial and follow-up surveys and disseminated the materials.

b Difference between study arms in participation and interest in dissemination at time of initial survey.

c Difference between study arms in participation and interest in dissemination at time of follow-up survey.

d Overall difference between study arms in participation and interest in dissemination.

**Table 3:** Participation in Dissemination of Materials, by Organization Type and Study Arm, Northern Appalachia Cancer Network *Screen for Life* Pilot Study, 2003

**Organization Type^a^**	**Total^b^No. (%)**	**Coalition Arm No. (%)**	**Noncoalition Arm No. (%)**
Civic, service, and fraternal	49 (29.2)^c^	37 (28.5)	12 (31.6)
Health care	42 (25.0)	34 (26.2)	8 (21.1)
Aging	37 (22.0)	31 (23.8)	6 (15.8)
Business	26 (15.5)	17 (13.1)	9 (23.7)
Religious	14 (8.3)^d^	11 (8.5)	3 (7.9)
Total	168 (100.0)	130 (100.0)	38 (100.0)

a Ninety organizations per type in each study arm were contacted for potential recruitment.

b Differences in the overall frequency distribution of organization type, *P* < .001.

c Civic, service, and fraternal organizations more likely to participate than business and religious organizations (*P* < .001).

c Religious organizations less likely to participate than civil, service, and fraternal; health care; and aging organizations (*P* < .001).

**Table 4:** Effect of Coalition Contact on Participation in Dissemination of Materials Within Coalition Study Arm, by Organization Type, Northern Appalachia Cancer Network *Screen For Life* Pilot Study, 2003

**Level of Influence**	**Total in Coalition Arm (N = 130)^a^ ^b^No. (%)**	**Type of Organization^b^**				

		**Civil, Fraternal, and Service (n = 37) No. (%)**	**Health Care (n = 34) No. (%)**	**Aging (n = 31) No. (%)**	**Business (n = 17) No. (%)**	**Religious (n = 11) No. (%)**
Strongly influenced	37 (31.1)	11 (31.4)	6 (20.7)	9 (31.0)	5 (31.3)	6 (60.0)
Somewhat influenced	49 (41.2)	16 (45.7)	12 (41.4)	11 (37.9)	8 (50.0)	2 (20.0)
Not very or not at all influenced	33 (27.7)	8 (22.9)	11 (37.9)	9 (31.0)	3 (18.8)	2 (20.0)

a Coalition contact was not found to have a significant effect on participation by organization type (*P = .*055).

b Eleven responses were missing (two from civil, fraternal, and service; five from health care; two from aging; one from business; and one from religious).

**Table 5:** Reasons for Dissemination of Materials, by Study Arm and Organization Type Within Coalition Arm, Northern Appalachia Cancer Network *Screen for Life* Pilot Study, 2003

**Reason^a^**	**Total (N = 168) No. (%)**	**Study Arm^b^**		**Organization Type in Coalition Arm**				

		**Coalition (n = 130) No. (%)**	**Noncoalition (n = 38) No. (%)**	**Civic, Fraternal, and Service (n = 37) No. (%)**	**Health Care (n = 34) No. (%)**	**Aging (n = 31) No. (%)**	**Business (n = 17) No. (%)**	**Religious (n = 11) No. (%)**
Promote health of community	120 (71.4)	90 (69.2)	30 (78.9)	25 (67.6)	27 (79.4)	19 (61.3)	10 (58.8)	9 (81.8)
Promote health of organization's clientele	108 (64.3)	83 (63.8)	25 (65.8)	19 (51.4)	30 (88.2)^c^	23 (74.2)	8 (47.1)	3 (27.3)
Promote health of organization's members	99 (58.9)	76 (58.5)	23 (60.5)	20 (54.1)	22 (64.7)	15 (48.4)	10 (58.8)	9 (81.8)
Mission of organization	72 (42.9)	60 (46.2)	12 (31.6)	14 (37.8)	27 (79.4)^d^	11 (35.5)	2 (11.8)	6 (54.5)
Community coalition research	70 (41.7)	57 (43.8)	13 (34.2)	19 (51.4)	16 (47.1)	13 (41.9)	5 (29.4)	4 (36.4)
Organization members' experience with cancer	42 (25.0)	22 (16.9)	4 (10.5)	3 (8.1)	11 (32.4)^e^	5 (16.1)	0 (0.0)	3 (27.3)

a Multiple reasons could be selected by each responding organization.

b Reasons for participation did not vary significantly between study arms.

c Compared with other organizations (except for aging), health care organizations were more likely to participate to promote the health of their clientel (*P* < .001)^c^, because it was the mission of the organization (*P* < .001)^d^, and because of their experience with cancer (*P = .*01)^e^.

d Compared with other organizations (except for aging), health care organizations were more likely to participate to promote the health of their clientele (*P* < .001)^c^, because it was the mission of the organization (*P* < .001)^d^, and because of their experience with cancer (*P = .*01)^e^.

e Compared with other organizations (except for aging), health care organizations were more likely to participate to promote the health of their clientele (*P* < .001)^c^, because it was the mission of the organization (*P* < .001)^d^, and because of their experience with cancer (*P = .*01)^e^.

**Table 6:** Initial and Change in Level of Interest and Importance of Dissemination, by Study Arm and Organization Type in Coalition Arm, Northern Appalachia Cancer Network *Screen for Life* Pilot Study, 2003

**Interest Level **	**Total (N = 168)^a^No. (%)**	**Study Arm**		**Organization Type in Coalition Arm**				

		**Coalition (n = 130) No. (%)**	**Noncoalition (n = 38) No. (%)**	**Civil, Fraternal, and Service (n = 37) No. (%)**	**Health Care (n = 34) No. (%)**	**Aging (n = 31) No. (%)**	**Business (n = 17) No. (%)**	**Religious (n = 11) No. (%)**

**Level of interest at start of study^b^**								

Very or somewhat interested	159 (97.5)	123 (97.6)	36 (97.3)	36 (100.0)	33 (100.0)	30 (96.8)	14 (87.5)	10 (100.0)
Not very or not at all interested	4 (2.5)	3 (2.4)	1 (2.7)	0 (0.0)	0 (0.0)	1 (3.2)	2 (12.5)	0 (0.0)

**Change in interest level during study^c^**								

Increased	1 (0.6)	1 (0.8)	0 (0.0)	0 (0.0)	0 (0.0)	0 (0.0)	1 (6.7)	0 (0.0)
Maintained	138 (89.6)	110 (90.2)	28 (87.5)	31 (88.6)	33 (100.0)	26 (86.7)	14 (93.3)	6 (66.7)
Decreased	15 (9.7)	11 (9.0)	4 (12.5)	4 (11.4)	0 (0.0)	4 (13.3)	0 (0.0)	3 (33.3)

**Level of importance at start of study^d^ **								

Very or somewhat important	150 (92.0)	117 (92.9)	33 (89.2)	34 (94.4)	33 (100.0)	30 (96.8)	11 (68.7)	9 (90.0)
Not very or not at all important	13 (8.0)	9 (7.1)	4 (10.8)	2 (5.6)	0 (0.0)	1 (3.2)	5 (31.3)	1 (10.0)

**Change in importance level during study^e^**								

Increased	4 (2.6)	3 (2.5)	1 (3.0)	1 (2.9)	0 (0.0)	0 (0.0)	2 (12.5)	0 (0.0)
Maintained	137 (90.1)	107 (89.9)	30 (90.9)	32 (94.2)	30 (100.0)	27 (90.0)	12 (75.0)	6 (66.7)
Decreased	11 (7.2)	9 (7.6)	2 (6.1)	1 (2.9)	0 (0.0)	3 (10.0)	2 (12.5)	3 (33.3)

a Columns may not equal N because of missing values.

b Initial interest did not differ significantly between study arms (*P = .*30) but did differ significantly between organization types in coalition arm (*P = .*02).

c Change in interest differed significantly between study arms (*P* < .001) and differed significantly between organization types in coalition arm (*P = .*01)

d Importance differed significantly between study arms (*P = .*03) and among organization types in coalition arm (*P = .*002).

e Change in importance did not differ significantly between study arms (*P = .*95) but did differ significantly between organization types in coalition arm (*P = .*006).
